# Implementing an Observation Protocol for Low-Risk Pediatric Head Trauma in a Community Hospital Setting to Reduce Patient Transfers

**DOI:** 10.51894/001c.144136

**Published:** 2025-09-30

**Authors:** Christopher Kilen

**Affiliations:** 1 Department of Emergency Medicine Henry Ford Wyandotte

## 41

### BACKGROUND

Pediatric head trauma is a common emergency department presentation. Its management varies in hospitals without pediatric specialists. At our Level 3 trauma center, we observed frequent transfers of low-risk pediatric head trauma patients. Transfers consume significant resources in a low-risk patient population. The PECARN decision rule has been well validated and in a low-risk patients the risk of significant intracranial pathology requiring intervention is less than 0.05% of cases.

### AIMS/OBJECTIVES

To develop a protocol in collaboration with the department of trauma surgery and the emergency department to observe low risk by PECARN pediatric head trauma activation patients and avoid transfer. A secondary aim was to evaluate the compliance and safety of the protocol.

### METHODS

A protocol to observe and reassess patients at fixed intervals and implemented in September of 2024. A retrospective chart review of all pediatric head injury trauma activations was preformed over an eight-month period from June 2024 through January of 2025. A total of 32 patients’ charts were reviewed for disposition and to evaluate patients’ PECARN risk.

### RESULTS

Of the 32 pediatric head trauma activations, 19 patients were transferred to a pediatric tertiary care center (59%). 8 of 12 (67%) patients were transferred prior to implementing the protocol. After protocol implementation, 11 of 20 (55%) patients were transferred to a pediatric tertiary care center. This 12% reduction was not statistically significant (p value 0.53) likely secondary to limited sample size. 7 of the 11 transferred patients were not low risk by the PECARN criteria and were excluded from the protocol due to risk. There were no clinically significant adverse events discovered on review.

### DISCUSSION

A 12% decrease in the transfer rate was found three months after implementation of an observation protocol for low-risk pediatric head trauma. Future enrollment may allow the protocol to reach significance. No adverse events were discovered in observing low risk by PECARN pediatric head trauma at community-based setting.

